# T cell-mediated immunodysregulation in multiple sclerosis: from pathogenic subsets to therapeutic advances

**DOI:** 10.3389/fimmu.2026.1740656

**Published:** 2026-04-15

**Authors:** Zhimei Wang, Donghe Han, Xikai Qiao, Xinyue Zhao, Chun Zhao, Shuangping Liu, Ran Tao, Meihua Jin, Peng Qu

**Affiliations:** 1 Department of Immunology, Medical College, Dalian University, Dalian, Liaoning, China; 2 Dalian University Affiliated Xinhua Hospital, Dalian, Liaoning, China; 3 Department of Anatomy, Medical College, Dalian University, Dalian, Liaoning, China; 4 Engineering Technology Research Center for the Utilization of Functional Components of Organic Natural Products, Dalian University, Dalian, Liaoning, China; 5 Institute of Marine Traditional Chinese Medicine, Dalian, Liaoning, China

**Keywords:** demyelination, disease-modifying therapy, immunocyte, neuron degeneration, traditional medicine

## Abstract

Multiple sclerosis (MS) is a demyelinating disease of the central nervous system (CNS) caused by chronic inflammation. It is the leading cause of neurologic symptoms in young people and leads to progressive neurodegenerative disability. Accumulating evidence indicates that MS arises from the coordinated and co-dominant actions of peripheral immune cells, meningeal tertiary lymphoid structures (TLS), and CNS-resident immune compartments. Within this complex immunopathological network, dysregulated T-cell-mediated adaptive immune responses play a pivotal role in initiating and organizing autoimmune inflammation. Peripherally activated T cells cross the blood-brain barrier (BBB), become reactivated within the CNS, and secrete pro-inflammatory cytokines that drive demyelination and neurodegeneration. Improved understanding of these immune mechanisms has led to the development of disease-modifying therapies (DMTs), many of which directly or indirectly target T-cell function. Here, we adopt a T-cell-centric perspective to systematically review the pathogenic mechanisms of MS, with particular emphasis on recent advances and unresolved questions regarding T-cell subset dysregulation, systematically integrating its precise targeting associations with conventional disease-modifying therapies (DMTs). Simultaneously, the mechanisms of emerging therapies were analyzed, and the potential of traditional herbal medicines was explored. This approach overcomes the limitations of previous studies that focused solely on a single T cell subset or a single therapeutic category.

## 1 Introduction

Multiple sclerosis (MS) is a chronic inflammatory demyelinating disease of the central nervous system (CNS) and a leading cause of nontraumatic disability in young adults aged 18–40 years (1). Globally, the prevalence of MS has increased by 30% since 2013 (2). Currently, 2.8 million people, with a 3:1 female-to-male ratio (2), are estimated to be living with this condition (World Health Organization, August 2023). There are four recognized clinical phenotypes of MS: relapsing-remitting MS (RRMS), secondary progressive MS (SPMS), primary progressive MS (PPMS), and clinically isolated syndrome (CIS). Contemporary disease classification frameworks increasingly emphasize integrating clinical course characteristics with key modifiers, including inflammatory activity and disability progression. Notably, robust evidence demonstrates that disability accumulation can occur independently of overt clinical relapses, a phenomenon formally designated “progression independent of relapse activity (PIRA)” (3–5). PIRA significantly contributes to long-term disability accumulation and can even be detected during relapse periods, highlighting the insidious and persistent nature of MS progression that transcends traditional subtype boundaries (6, 7). The pathogenesis of MS is driven by peripheral immune activation and complex interactions between meningeal peripheral lymphoid structures (TLS) and CNS resident immune cells. Once within the CNS, these activated immune cells trigger an inflammatory cascade, leading to destruction of the myelin sheath (the protective covering around nerve fibers) and the formation of sclerotic plaques (8, 9). This results in symptoms that can range from motor deficits (10) and sensory deficits (11) to bladder dysfunction (12) or cognitive decline (13), with accumulating adverse effects on the physical and psychosocial aspects of the patient’s functioning and quality of life (14).

Although other immune cells-particularly B cells and innate immune populations within the CNS-play indispensable roles in MS pathogenesis, this review adopts a T-cell-focused perspective. Rather than excluding other immune contributors, this approach aims to establish a coherent mechanistic axis that integrates immunopathogenesis with therapeutic intervention. Accordingly, this review adopts a T-cell-centric framework to systematically analyze the dysregulation of major T-cell subsets in MS and their precise targeting by existing and emerging therapeutic strategies. By comparing conventional DMTs with novel therapeutic approaches and exploring the immunomodulatory potential of traditional herbal medicines, this review aims to provide an integrated “mechanism-therapy-translation” perspective to inform future therapeutic development. While acknowledging the significant role of other immune cells, including B cells, within the CNS, this review will initially focus on T cells as a theoretical foundation for integrating immunopathogenesis and therapeutics.

While several recent reviews have summarized the immunology or therapeutic landscape of MS, many have addressed these aspects separately. In contrast, the present review aims to integrate advances in T-cell subset biology with the mechanisms of current and emerging therapies within a unified framework. By linking pathogenic T-cell programs to distinct modes of therapeutic intervention, this review seeks to highlight how different treatment strategies converge on the regulation of autoreactive T-cell-driven immune networks. In doing so, we aim to provide a conceptual bridge between immunopathogenesis and therapeutic strategy within the evolving MS literature.

## 2 Immunopathogenesis of MS

In the pathological cascade of MS, the central pathogenic mechanism is an autoimmune inflammatory response driven by the activation of autoreactive T cells and B cells. This process is critically amplified by disruption of the BBB, which permits infiltration of peripheral immune cells into the CNS and thereby exacerbates demyelination (1, 15–17). Concurrently, T cells, B cells, and dendritic cells within the meningeal and subarachnoid spaces can organize into ectopic lymphoid structures-termed meningeal TLS-that function analogously to secondary lymphoid organs. These TLS serve as local immunological hubs, orchestrating antigen-driven B- and T-cell responses, including clonal expansion and antibody production (18, 19). For example, single-cell RNA sequencing of cerebrospinal fluid (CSF) from MS patients has identified intrathecal enrichment of B cells, follicular helper T (Tfh) cells, and T helper 17 (Th17) cells. Cytokines secreted by these cells-including IL-17-have been implicated in both the formation and functional maintenance of TLS (20–22). Notably, in patients exhibiting a high burden of cortical lesions, CSF analysis consistently reveals elevated levels of pro-inflammatory cytokines (e.g., IFN-γ, TNF), chemokines (e.g., CXCL13), B-cell survival factors (e.g., BAFF), and biomarkers of BBB dysfunction (e.g., fibrinogen, complement components), all of which collectively intensify the local neuroinflammatory milieu (23–25). Meanwhile, CNS-resident microglia and astrocytes, upon activation, sustain chronic release of diverse inflammatory mediators (26–32). These endogenous signals synergize with infiltrating and locally activated lymphocytes to establish a self-perpetuating inflammatory circuit. This cycle not only drives progressive demyelination but also actively suppresses remyelination, thereby underpinning the relentless clinical progression of MS (16, 33–35).

Extensive evidence indicates that the adaptive immune response-orchestrated primarily by pathogenic T cell-constitutes a pivotal driver of early neuropathological events in MS. Throughout disease progression, T cells undergo antigen-dependent activation and functional differentiation, subsequently infiltrating the CNS. Within the CNS, they engage in dynamic, multifaceted interactions with B cells, macrophages, neutrophils, and resident glial cells, collectively sustaining neuroinflammation and driving tissue damage. Collectively, these insights underpin our T-cell-centric conceptual framework for this review. However, it is essential to acknowledge that while the experimental autoimmune encephalomyelitis (EAE) model has served as a foundational tool for investigating T-cell-mediated mechanisms in demyelinating disease, its translational relevance remains constrained by several well-documented limitations. These include the artificial mode of disease induction (e.g., immunization with exogenous myelin antigens in adjuvant), the predominantly acute and monophasic nature of the inflammatory response, the atypical anatomical distribution of lesions, and discrepancies in the relative contributions of specific T-cell subsets compared with human MS. Notably, EAE represents an externally triggered, antigen-driven inflammatory model, whereas MS arises from a complex interplay of intrinsic (e.g., genetic, epigenetic) and extrinsic (e.g., environmental, infectious) factors. This fundamental divergence in etiopathogenesis helps explain the frequent failure of therapeutics demonstrating robust efficacy in EAE to replicate comparable clinical benefits in human trials. Consequently, precise identification and validation of mechanistically relevant, therapeutically tractable targets within this intricate immunological network remain a critical priority.

Research into the pathogenesis of MS indicates that helper T-cell subsets-including Th1, Th2, Th17, and regulatory T cells (Tregs)-are currently regarded as the most critical T lymphocyte populations involved in disease development. Moreover, other T-cell subsets, such as Th1, Th17, Tregs, Th2, Th9, Th22, and follicular helper T cells (Tfh), as well as pro-inflammatory and cytotoxic CD8^+^ T cells, also play indispensable roles (36–42).

### 2.1 Th1/Th2 cells

**Figure 1:**
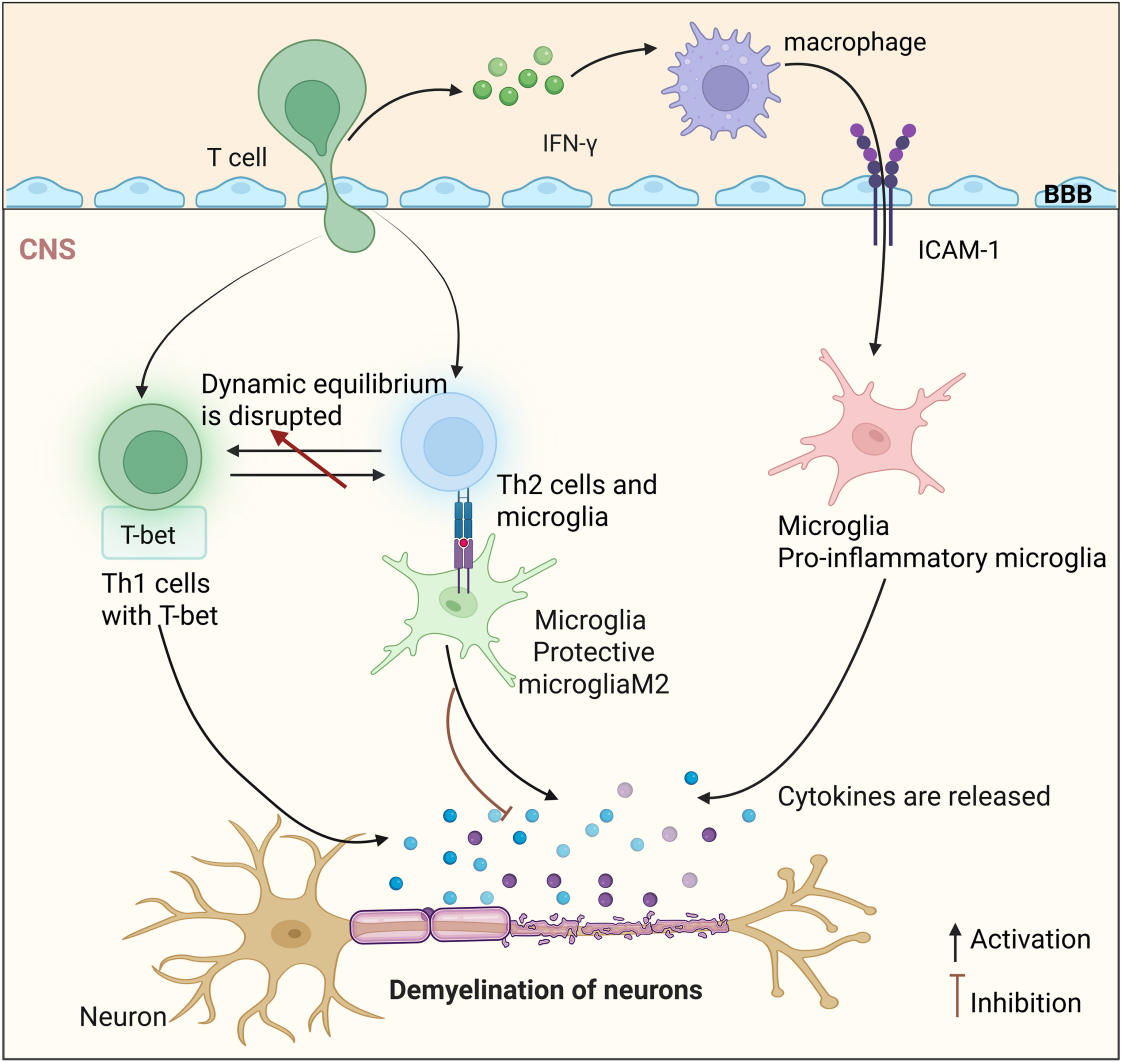
Disruption of the Th1/Th2 cell balance induces CNS inflammation. Activated T cells cross the blood-brain barrier (BBB) via intercellular adhesion molecule-1 (ICAM-1) and contribute to the activation of pro-inflammatory microglia and macrophages. Within the central nervous system (CNS), T helper 1 (Th1) cells, characterized here by the Th1-associated transcription factor T-bet, release interferon-γ (IFN-γ) and other mediators that promote inflammatory damage and demyelination. In contrast, T helper 2 (Th2)-associated responses may produce anti-inflammatory cytokines such as interleukin-10 (IL-10), supporting protective microglial states and limiting myelin injury. The balance between these immune programs is dynamic and context-dependent rather than linear. The schematic depicts proposed interactions based on available human and experimental evidence rather than a fixed temporal sequence.

Under physiological conditions, the human body maintains a dynamic equilibrium between Th1 and Th2 lymphocytes. In active MS, the Th1-polarized immune response is amplified. Interferon-gamma (IFN-γ), which is secreted by Th1 cells, activates macrophages and promotes their migration into the CNS via intercellular adhesion molecule 1 (ICAM-1)/intercellular adhesion molecule 2 (ICAM-2)-mediated adhesion. This process exerts pro-inflammatory effects (**Figure 1**). Consistently, IFN-γ levels are elevated in the systemic circulation, CSF, and CNS lesions of MS patients (40, 43–45). Molecular investigations suggest that the T-cell co-stimulatory molecule ADAM12 may modulate IFN-γ expression through T-bet-dependent mechanisms, thereby potentiating the Th1-driven inflammatory response (46). However, this observation is derived primarily from limited experimental data, and the generalizability and functional relevance of ADAM12 in human MS must be rigorously validated. Notably, direct functional evidence demonstrating that ADAM12 facilitates CNS infiltration of T cells is currently lacking. Recent studies have redefined the role of IFN-γ in MS pathogenesis, revealing that it is not a uniformly pathogenic mediator. In resident CNS cells, particularly astrocytes, IFN-γ signaling induces the expression of immunoproteasome subunits and programmed death ligand 1 (PD-L1). This mitigates oxidative stress and restrains chronic neuroinflammation. These findings reveal the context-dependent immunoregulatory functions of IFN-γ (47–49).

Furthermore, studies have demonstrated that dysregulated granulocyte-macrophage colony-stimulating factor (GM-CSF) expression in peripheral helper T cells can drive the spontaneous infiltration of myeloid cells into the CNS, thereby triggering severe immune-mediated pathological damage (50). In Th1 cells, the transcription factor T-bet promotes GM-CSF production by upregulating RUNX3, facilitating the development of chronic encephalitis (51). A distinct pathogenic subset of GM-CSF-producing helper T cells that co-expresses canonical Th1 and Th17 lineage markers has been identified. Its pathogenic function critically depends on T-bet activity. This finding reveals an independent GM-CSF-centered effector program underlying CNS autoimmunity (52).

The Th2-type immune response persists throughout MS and secretes anti-inflammatory cytokines, including IL-4 and IL-10, which suppress the proinflammatory activation of microglia and macrophages. This fosters a neuroprotective microenvironment and mitigates CNS tissue damage (53, 54) (**Figure 1**). Elevated levels of microRNAs (miRNAs) miR-210-3p and miR-544a have been observed in MS patients. These miRNAs disrupt the Th1/Th2 balance by targeting RUNX3. This results in an IFN-γ/IL-4 imbalance, contributing to CNS inflammation (55). EAE studies further indicate that Th17 cells exacerbate neuroinflammation by traversing the BBB via IL-17A- and CCR6-dependent mechanisms (56, 57). Despite the substantial potential of microRNAs in regulating T-cell differentiation and function, direct evidence linking specific microRNAs to Th1/Th2 imbalance in human MS remains limited. Consequently, related conclusions should be interpreted with appropriate caution.

### 2.2 Th17/Tregs

**Figure 2:**
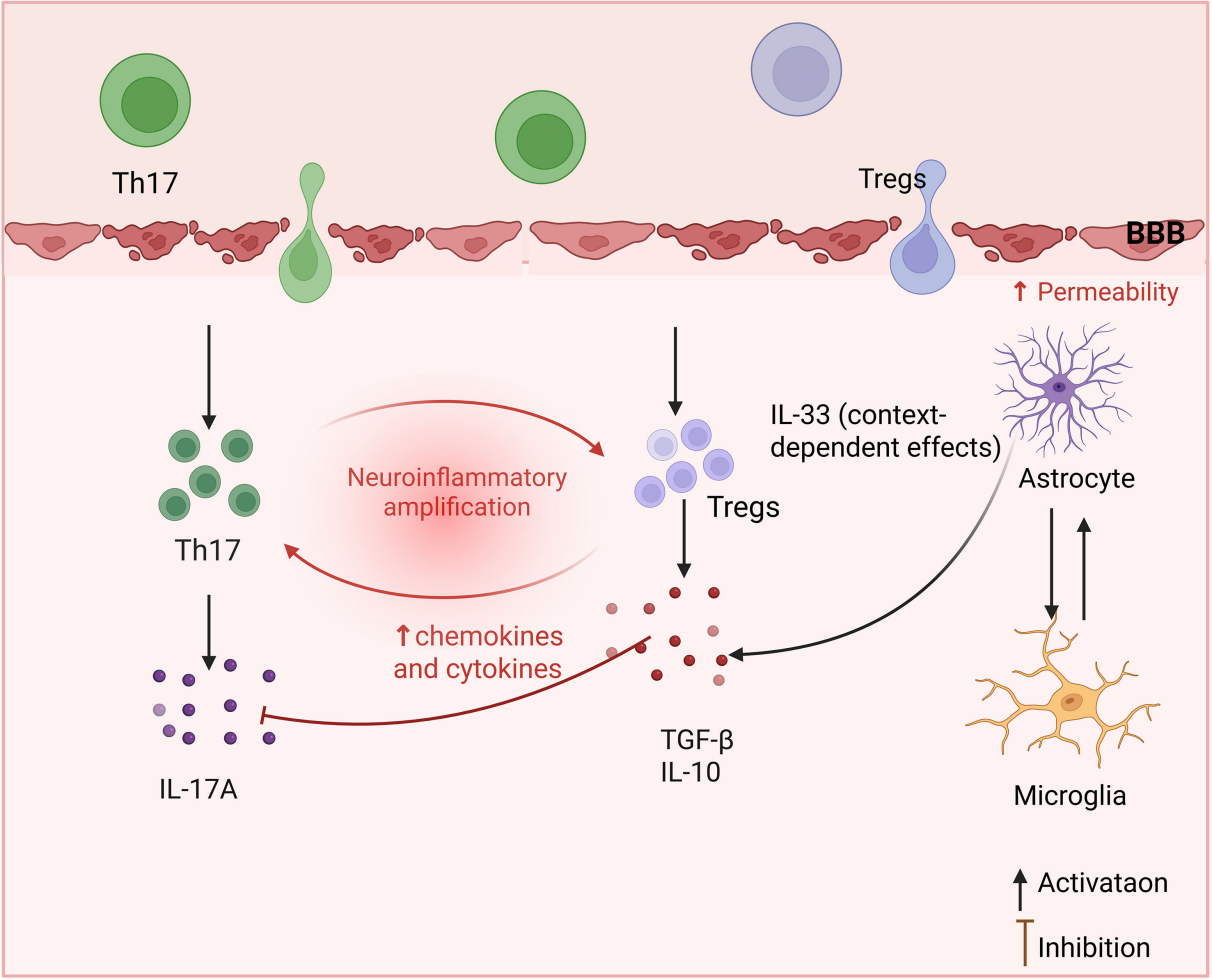
The effect of imbalance between Th17 and Tregs on the pathogenesis of MS. Disruption of the T helper 17 (Th17) cell/regulatory T cells (Tregs) equilibrium contributes to neuroinflammation within the central nervous system (CNS). Pathological immune activation promotes Th17 cell infiltration across the blood-brain barrier (BBB) and the release of interleukin-17A (IL-17A) and other pro-inflammatory mediators, thereby increasing BBB permeability and activating microglia and astrocytes. Reciprocal interactions between these glial cell populations contribute to neuroinflammatory amplification within the CNS. Concurrently, impaired Treg function, characterized by reduced production of transforming growth factor-β (TGF-β) and interleukin-10 (IL-10), weakens immunoregulatory and neuroprotective responses. Astrocyte-derived interleukin-33 (IL-33), which exerts context-dependent immunomodulatory effects, may support Treg activity and partially counteract CNS inflammation.

The imbalance between Th17 and Tregs plays a pivotal role in the pathogenesis of MS (**Figure 2**) (58, 59). Th17 cells have been demonstrated to play a dual role in tissue homeostasis and pro-inflammatory responses (60, 61). IL-17A, secreted by Th17 cells, has been shown to induce oligodendrocyte apoptosis, axonal degeneration, and neuronal dysfunction, thereby accelerating disease progression in EAE. Th17 cells exhibit significant functional heterogeneity and plasticity. In the presence of inflammatory conditions, these cells have been observed to downregulate RORγt and IL-17A expression, undergoing a process of differentiation into a state designated as “ex-Th17” cells. These “ex-Th17” cells are characterized by an augmented pro-inflammatory capacity, evidenced by increased secretion of IFN-γ, TNF, and GM-CSF (62–64). Furthermore, upon stimulation by IL-23 and other microenvironmental cues, Th17 cells acquire pathogenic effector programs involving GM-CSF and/or IFN-γ production, thereby evolving into critical encephalitogenic T cells in both MS and EAE (65–67). In accordance with this observation, GM-CSF levels have been shown to be markedly elevated in the cerebrospinal fluid and serum of MS patients (68, 69). Th17 cell migration into the CNS is mediated by multiple chemokine receptors, adhesion molecules, and integrins (70). Th17 cells infiltrate the CNS by engaging adhesion molecules, including DICAM, integrin αvβ3, and VLA-3, to traverse the BBB (71–73). Furthermore, IL-17A compromises BBB integrity by inducing reactive oxygen species (ROS) and matrix metalloproteinases (MMPs), thereby facilitating immune cell infiltration (74, 75). Furthermore, Th17 cells engage in synergistic interactions with B cells, astrocytes, and other CNS-resident or infiltrating immune cells, thereby further amplifying neuroinflammatory cascades (76, 77).

Tregs have been shown to maintain immune tolerance through the secretion or expression of key immunomodulatory molecules, including IL-10, IL-33, TGF-β, CTLA-4, and CD25 (78, 79). It is important to note that IL-33 displays a dual functional role, meaning that its effects can be either protective or pathogenic, depending on the disease stage and microenvironmental context (80, 81). Furthermore, Tregs have been shown to upregulate genes associated with M2-type macrophage polarization, thereby facilitating tissue repair and resolution of inflammation (79, 82). In patients diagnosed with MS, Tregs function is significantly impaired, as evidenced by reduced overall numbers, altered subset composition, diminished migratory capacity, and compromised suppressive activity (83, 84). Transcriptomic and epigenomic analyses reveal that pre-activation of the AP-1/IRF signaling axis induces preferential expression of the short isoform of PRDM1, which destabilizes FOXP3 expression and consequently undermines Tregs lineage stability and function (85). Importantly, Tregs cell functionality is subject to stringent regulation by metabolic and epigenetic mechanisms. Pro-inflammatory conditions have been shown to induce metabolic dysregulation, which in turn impairs mitochondrial integrity and bioenergetics (86, 87). Additionally, aberrant DNA methylation patterns have been observed to further constrain their immunosuppressive capacity (88). Collectively, these findings suggest that Tregs dysfunction reflects a multilayered, context-dependent dysregulation of regulatory mechanisms. Collectively, the pathogenic skewing toward pro-inflammatory Th17 responses, coupled with intrinsic Tregs dysfunction, constitutes a central pathological axis in MS.

### 2.3 Other T-cell subtypes

Under inflammatory conditions, a subset of Th17 cells can acquire a Th1-like phenotype, co-expressing IL-17A and IFN-γ, thereby exhibiting enhanced pathogenicity (89, 90). These cells secrete multiple pro-inflammatory cytokines, including TNF-α and GM-CSF, promoting the recruitment of brain-derived T cells within the CNS and exacerbating neuroinflammation (77, 91).

Circulating Tfh cell subsets (e.g., Tfh1, cTfh17, cTfh17.1) are increased in MS patients and infiltrate the CNS (92–95). Recent evidence highlights the role of follicular regulatory T (Tfr) cells, which suppress germinal center activity through the release of anti-inflammatory cytokines (IL-10 and TGF-β1) (96). Reduced Tfr/Tfh ratios in serum and CSF of MS patients drive pathological IgG production, thereby promoting CNS autoimmunity in MS (94, 97). Treatment with rituximab (RTX) in MS patients has been found to reduce cTfh cells and serum IL-21 levels, improving clinical symptoms (98).

The Th9 cell subset, characterized by IL-9, IL-10, and IL-21 secretion, appears to exert anti-inflammatory effects in MS models. IL-9 administration significantly ameliorates clinical symptoms in EAE mice, suggesting potential immunomodulatory properties (99, 100). Supporting this possibility, treatment with a histamine H4 receptor antagonist reduces IL-9 and IRF4 mRNA levels in CNS tissues while improving EAE outcomes (101). However, the role of IL-9 in MS remains controversial, with studies suggesting that IL-9 may act as a pro-inflammatory factor in the development of EAE (102). Therefore, the function of IL-9 in MS may depend on the disease context or environment.

**Figure 3:**
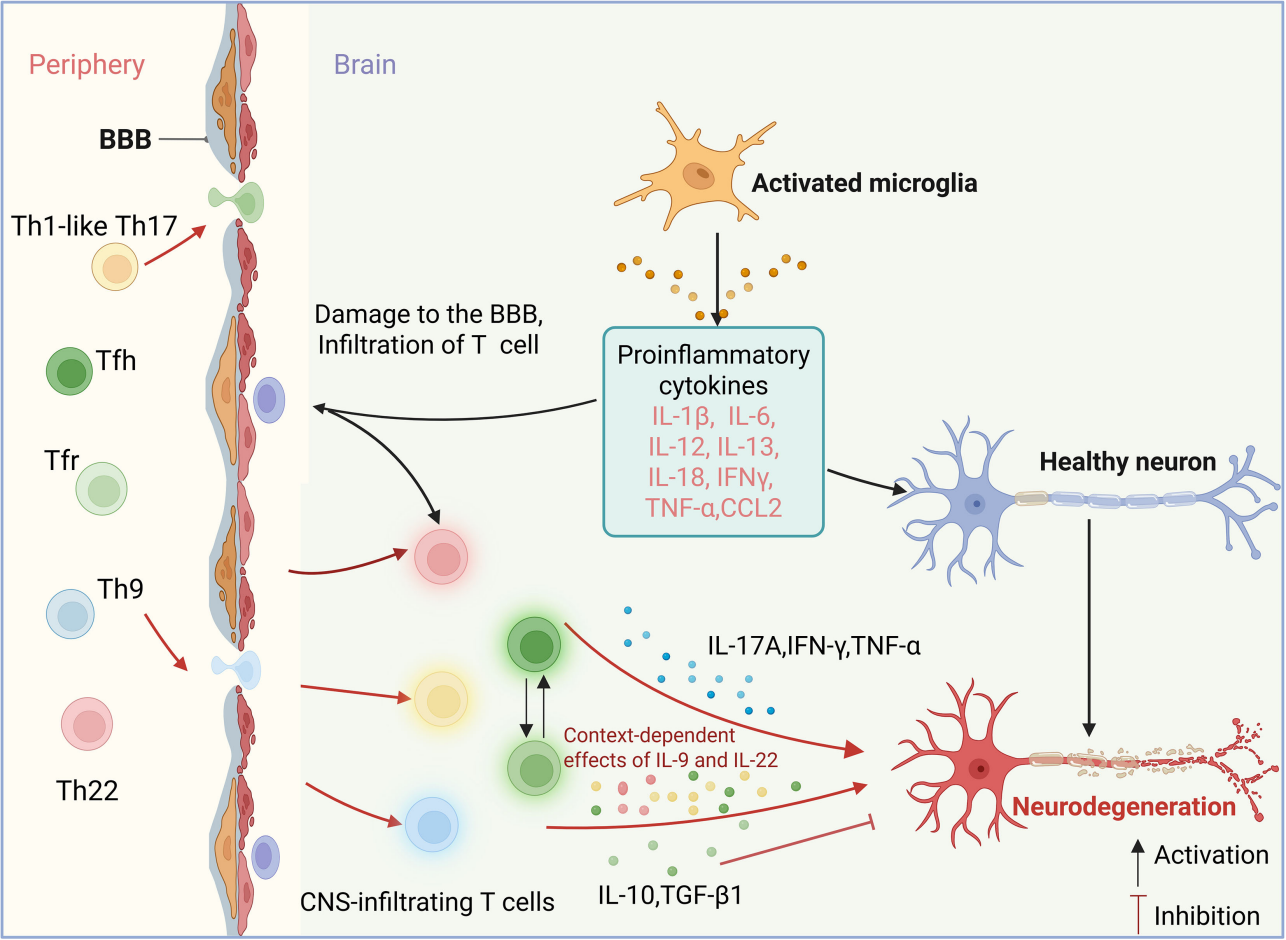
Mechanisms of selected non-classical CD4^+^ T-cell subsets in the pathogenesis of MS. Peripheral immune dysregulation promotes the migration of T helper 1-like T helper 17 cells (Th1-like Th17 cells), follicular helper T cells (Tfh), follicular regulatory T cells (Tfr), T helper 9 cells (Th9), and T helper 22 cells (Th22) across the blood-brain barrier (BBB) into the central nervous system (CNS). Cytokines secreted by these cells, including interleukin-17A (IL-17A), interferon-γ (IFN-γ), interleukin-9 (IL-9), interleukin-22 (IL-22), and tumor necrosis factor-α (TNF-α), may activate microglia and astrocytes, thereby promoting neuroinflammation. Activated glial cells release pro-inflammatory mediators that contribute to myelin damage and neurodegeneration. In addition, IL-9 and IL-22 have been reported to exert context-dependent protective effects, including regulation of regulatory T-cell (Treg) responses and maintenance of blood-brain barrier integrity. Dysregulation of the Tfh/Tfr axis further alters the balance between interleukin-21 (IL-21) and interleukin-10 (IL-10) signaling.

The role of Th22 cells in MS remains less clear; however, mechanistic studies have implicated IL-22 in MS pathogenesis. Treatment with a cathepsin B inhibitor attenuated EAE severity concurrent with reduced IL-22 levels, suggesting Th22-mediated inflammatory effects (**Figure 3**) (103). However, an intriguing experiment revealed that the absence of IL-22 antagonists resulted in milder EAE manifestations, suggesting that IL-22 may exert a potential protective effect on neuroinflammatory processes (104).

CD8^+^ T cells predominate in active MS lesions and exhibit pronounced oligoclonal expansion across the CNS, CSF, and peripheral blood-consistent with antigen-driven cytotoxic responses (105, 106). In SPMS, a substantial proportion of lesion-associated CD8^+^ T cells adopt a tissue-resident memory (T_RM)-like phenotype (e.g., CD69^+^, CD103^+^/^-^, CCR7^-^), accumulating preferentially in the perivascular space and potentially sustaining compartmentalized CNS inflammation (107). Demyelination-induced upregulation of MHC-I on neurons may further facilitate CD8^+^ T cell-mediated axonal injury (108). Additionally, GM-CSF-producing CD8^+^ T cell subsets have been implicated in amplifying myeloid cell–driven neuroinflammation within the CNS (69).

Collectively, multiple T-cell subsets contribute to the pathogenesis of MS through distinct cytokines. The presence of pathogenic Th17/Th1-like cells has been observed to co-produce IL-17A and IFN-γ, along with TNF-α and GM-CSF, thereby amplifying CNS inflammation. Expanded Tfh subsets (Tfh1, cTfh17, cTfh17.1) have been shown to promote IL-21-mediated B-cell activation, while reduced Tfr-derived IL-10 and TGF-β1 have been observed to disrupt germinal center restraint, thereby favoring pathological IgG production. Th9 cells secrete IL-9, IL-10, and IL-21, exerting context-dependent immunomodulatory effects. Th22-derived IL-22 demonstrates a dual capacity, exhibiting both inflammatory and protective properties. Clonally expanded CD8^+^ T cells, including T_RM-like populations, have been shown to mediate antigen-driven toxicity and GM-CSF-associated myeloid activation, contributing to axonal injury and compartmentalized neuroinflammation.

### 2.4 Age-dependent T-cell remodeling in MS

Age has been identified as a pivotal factor in the clinical progression of MS (109). As people age, the thymus gland begins to function less efficiently, and the population of naïve T cells decreases. This decline contributes to the acceleration of immune system senescence and increased susceptibility to MS in older adults (110, 111). Analyses of peripheral blood samples from MS patients have revealed an abnormal, age-related expansion of circulating CD4^+^ T cells in elderly MS patients. This phenomenon is potentially contributing to sustained, compartmentalized immune responses in the CNS and subsequent disease progression (112). Conversely, the numbers of both B cells and CD4^+^ T cells in MS patients decline with age, suggesting an age-dependent reduction in immune cell infiltration into the CSF, particularly in patients with PPMS. For instance, Th17 cells and the pro-inflammatory cytokine IL-17 exhibit age-associated decreases, especially among female MS patients (113). Notably, young MS patients exhibit indications of premature immunosenescence, particularly within the CD8^+^ T-cell compartment, which may contribute to disease severity (114). Consequently, elucidating the age-dependent dynamics of immune cell subsets will facilitate the development of more precise, age-stratified therapeutic strategies for MS.

In summary, effective prevention and treatment of MS necessitate a more profound understanding of the mutual regulatory mechanisms among distinct T-cell subsets, as well as rigorous validation of the safety and efficacy of T-cell-targeted therapies.

## 3 Immunotherapy for MS

### 3.1 Immunomodulation against T cells

DMTs have become the cornerstone for reducing inflammatory activity and delaying clinical progression. Current DMTs target distinct immunopathological steps, including lymphocyte activation and differentiation, leukocyte trafficking across the BBB, oxidative stress, and broader immune reconstitution. Accordingly, therapeutic strategies range from first-line injectable agents (e.g., interferon-β and glatiramer acetate) to oral small molecules (e.g., teriflunomide, dimethyl fumarate, and S1P receptor modulators) and high-efficacy monoclonal antibodies (e.g., natalizumab, alemtuzumab, and anti-CD20 therapies). In the following sections, we summarize the major mechanisms of action, clinical efficacy, and safety considerations of these representative DMT classes to provide an integrated framework for understanding current MS treatment paradigms (**Table 1**).

**Table 1:** Mechanism-based classification of DMTs for MS.

DMT class	Drug	Primary target/mechanism	Affected immune cells	Key adverse events	Approval	Citations
Immune deviation therapy	IFN-β	Inhibits antigen presentation & Th1 activation;modulates cytokines;JAK/STAT-related effects	↓ Th1 and Th17 cells; ↑ regulatory immune responses	Flu-like symptoms; depression; elevated liver enzymes; leukopenia/anemia	FDA, Berlex, 1993	(115–121, 126–128)
	Glatiramer acetate	Induces Th2 immune deviation; competitively binds MHC II to block myelin antigen presentation	↑ Th2 cells; ↑ Tregs; modulation of B-cell antigen presentation	Injection-site reactions	FDA, Teva, 1996	(117, 146–159, 161, 162)
Metabolic and redox modulation	Teriflunomide	Inhibits DHODH, suppressing *de novo* pyrimidine synthesis and lymphocyte proliferation	↓ proliferating CD4^+^ T cells; functional enhancement of Tregs	Elevated liver enzymes; hypertension; hair loss; diarrhea	FDA, Sanofi, 2012	(146, 163, 164, 169–172, 175–178)
	Dimethyl fumarate	Activates NRF2 antioxidant pathway;	↓ Th17 and effector memory T cells; ↑ naïve T cells	Flushing; gastrointestinal distress; lymphopenia (rare PML risk)	FDA, Biogen, 2013	(179–195)
Cell migration inhibition	Natalizumab	Blocks α4-integrin-VCAM-1 interaction, preventing immune cell entry into the CNS	↓ CD4^+^ and CD8^+^ T cells in CNS/CSF (via trafficking blockade)	Progressive multifocal leukoencephalopathy; infections	FDA, Biogen, 2004	(129, 136, 138, 139, 143–145)
Lymphocyte trafficking modulation	Fingolimod	Functional antagonism of S1P receptors via receptor internalization (requires phosphorylation)	↓ circulating T and B cell	Bradycardia; lymphopenia; elevated liver enzymes; macular edema; infections	FDA, Novartis, 2010	(196–209)
	Siponimod	Selective S1P1/S1P5 modulation	↓ circulating T cell	Bradycardia; macular edema; infections	FDA, Novartis, 2019	(196–209)
	Ozanimod	Selective S1P1/S1P5 modulation	↓ circulating lymphocytes	Elevated liver enzymes; infections	FDA, Bristol Myers Squibb, 2020	(196–209)
	Ponesimod	Selective S1P1 modulation	↓ circulating lymphocytes	Hypertension; bradyarrhythmia; infections	FDA, Janssen, 2021	(196–209)
Immune reconstitution therapy	Alemtuzumab	Anti-CD52-mediated lymphocyte depletion via CDC and ADCC, followed by immune reconstitution	↓ T and B cells followed by immune reconstitution; ↑ Tregs	Severe infections; secondary autoimmunity; long-term monitoring required	FDA, Sanofi, 2014	(210–221)
	Cladribine	DNA damage-induced apoptosis following intracellular phosphorylation	↓ CD4^+^ and CD8^+^ T cells (preferentially memory subsets); ↓ CD19^+^ B cells	Lymphopenia; malignancy risk; infections; bone marrow suppression	EMA, Merck, 2017; FDA, Merck, 2019	(222–239)

↑ increase, ↓ decrease.

#### 3.1.1 IFN-β

**Figure 4:**
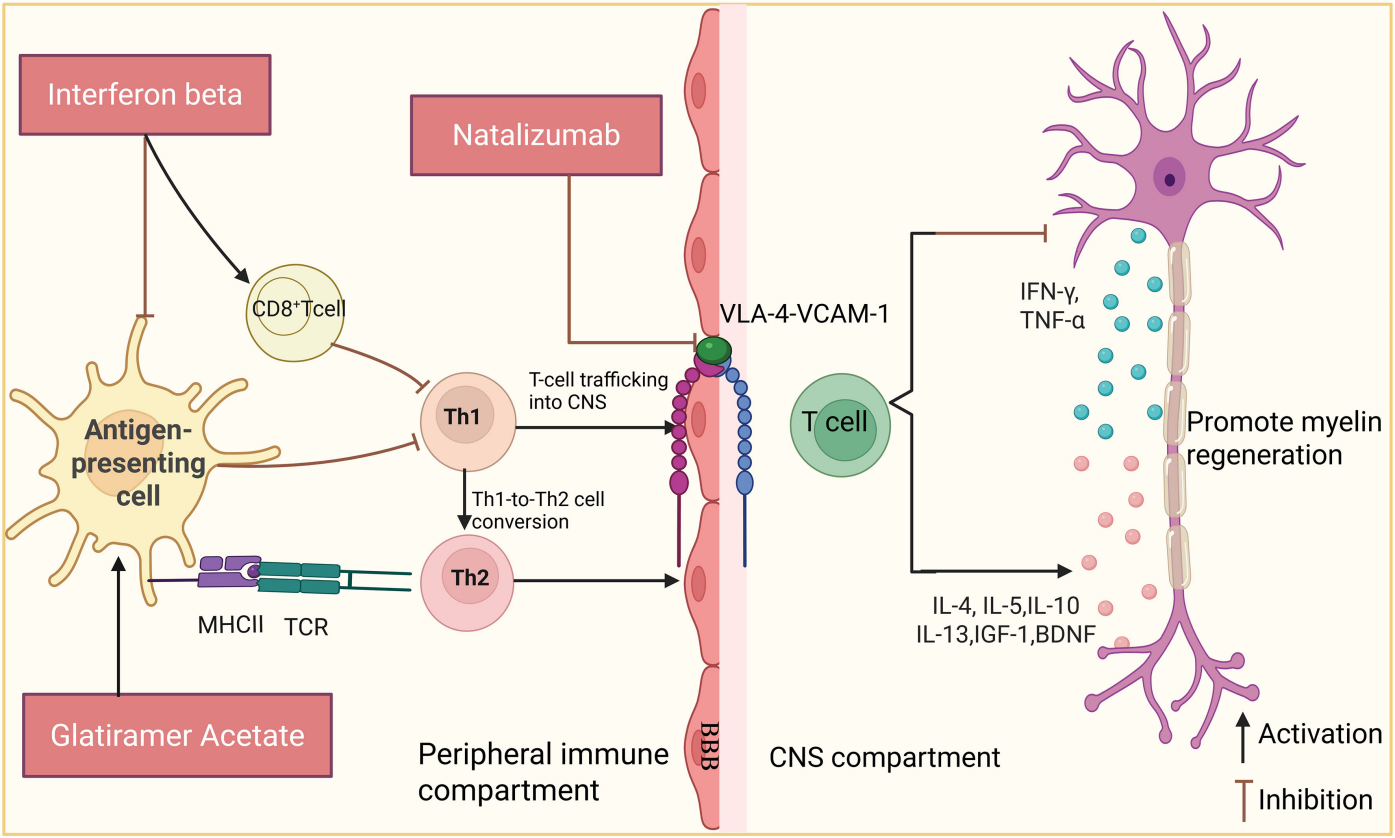
Pharmacological treatments targeting T-cell immunomodulation--IFN-β, GA, NAT. IFN-β reduces antigen-presenting cell activity and downstream pro-inflammatory cytokine production, thereby limiting T helper 1 (Th1)-associated inflammatory responses and favoring anti-inflammatory T-cell programs. GA interferes with myelin antigen presentation through major histocompatibility complex class II (MHC class II) molecules and T-cell receptor (TCR)-dependent signaling, promotes a shift from Th1- toward T helper 2 (Th2)-associated responses, and enhances production of anti-inflammatory or neuroprotective mediators, including interleukin-4 (IL-4), IL-5, IL-10, IL-13, insulin-like growth factor-1 (IGF-1), and brain-derived neurotrophic factor (BDNF). Natalizumab (NAT) blocks interactions between very late antigen-4 (VLA-4) and vascular cell adhesion molecule-1 (VCAM-1) at the blood-brain barrier (BBB), thereby preventing pathogenic T-cell trafficking into the central nervous system (CNS). The schematic also indicates reduced production of interferon-γ (IFN-γ) and tumor necrosis factor-α (TNF-α), together with enhanced neuroprotective signaling. These mechanisms are associated with reduced relapse rates and decreased magnetic resonance imaging (MRI) lesion activity, with therapy-specific safety considerations.

Interferon beta (IFN-β) is a class-I interferon, the first class of disease-modifying agents for the treatment of MS (**Figure 4**). In 1993, IFN-β1a became the first of these therapeutic agents approved by the Food and Drug Administration (FDA) for use in patients with MS (115). The four IFN-β drugs currently approved in the US and EU are subcutaneous (SC) IFN-β-1b, SC IFNβ-1a, intramuscular injection of IFN-β-1a, and SC polyethylene glycol interferon beta-1a (116).

IFN-β can limit the entry of T cells into the CNS by decreasing the antigen presentation capacity and, thus, the stimulation of Th1 cells. This further decreases the production of the pro-inflammatory cytokines IFN-γ and IL-12 and induces the production of anti-inflammatory cytokines IL-4, IL-5, and IL-10 (117, 118) (**Figure 4**).

Beyond the classical mechanisms described above, increasing attention has been directed toward additional immunomodulatory effects of IFN-β. Accumulating evidence indicates that IFN-β signaling suppresses the differentiation and pathogenicity of Th17 cells (119), potentially by attenuating IL-27-driven pathogenic Th17 responses, thereby indirectly restraining GM-CSF-associated effector programs and limiting CNS inflammation (120, 121). In addition to its direct effects on adaptive immunity, IFN-β also modulates innate immune pathways by reshaping myeloid cell activation states and pro-inflammatory cytokine profiles, which indirectly constrains T-cell reactivation within the CNS (122). Furthermore, recent clinical studies have shown that the activity of brain-reactive peripheral B cells can predict clinical responsiveness to IFN-β therapy in MS patients, with specific B-cell reactivity profiles being associated with improved therapeutic outcomes (123). These findings suggest that B-cell-associated immune states contribute to the modulation of IFN-β efficacy.

Patients with SPMS treated with IFN-β experience substantially reduced production of pro-inflammatory factors and enhanced expression of (antioxidant) metallothionein genes (124). IFN-β also enhanced the function of CD8^+^ T cells, while CD8^+^ T cells inhibited the autoreactivity of CD4^+^ T cells, thereby reducing the MS recurrence rate by 30% and mitigating patient progression to clinically diagnosed MS (125). Recent studies have reported the possibility that the positive effects of IFN-β in RRMS patients may be mediated by restoring regulatory noncoding RNA-20b (miR-20b) expression to normal levels through the janus kinase-signal transducer and activator of transcription signaling pathway (126). A double-blind study involving 301 patients with MS revealed that treatment using interferon beta significantly reduced expanded disability status scale (EDSS) scores compared to an untreated control group, while markedly improving clinical symptoms (127). Additionally, it is important to note that IFN-β may offer only modest benefits for SPMS patients with ongoing active relapses, while it is largely ineffective for those in the purely progressive phase without relapses. It does not significantly slow neurodegeneration or disability progression. Furthermore, it carries certain side effects, primarily including flu-like symptoms, depression, elevated liver enzymes, leukopenia, or anemia (124, 128).

#### 3.1.2 NAT

Natalizumab (NAT) is a humanized IgG4 antibody approved by the FDA in 2004 for the treatment of MS (129). It specifically recognizes α4 integrin (also known as the α4 subunit of VLA-4, or CD49d) expressed on the surface of activated T cells (130, 131). Binding of α4 integrin to its ligand, vascular cell adhesion molecule-1 (VCAM-1), mediates the adhesion and migration of immune cells to endothelial cells (132) (**Figure 4**). NAT exerts its therapeutic effect by blocking this interaction, thereby inhibiting the migration of immune cells into CNS tissues (133, 134). Furthermore, Niino et al. were the first to demonstrated that B cells express significantly higher levels of the surface target molecule VLA-4 compared to T cells, thereby inhibiting the entry of pathogenic B cells into the CNS. Correspondingly, NAT effectively blocks their ability to migrate through brain endothelial cells *in vitro* (135, 136). Evidence further suggests that short-term “pretreatment” with NAT during the early stages of MS can trap pathogenic T-cell populations within peripheral tissues amenable to intervention. Subsequent specific elimination of these cells holds promise for halting disease progression (137).

Analysis of CSF from MS patients undergoing NAT treatment revealed significantly lower numbers of CD4^+^ T cells and CD8^+^ T cells compared to untreated MS patients (138, 139). In studies examining the effects of a single dose of NAT administered shortly after a relapse, early stabilization or improvement in EDSS scores was observed, suggesting a rapid onset of therapeutic activity (140, 141). Additionally, the use of NAT in treating MS patients revealed that its effects on peripheral immune cells and pharmacodynamic markers are reversible, enabling timely adjustments to treatment strategies (142). Recent studies indicate that NAT effectively suppresses inflammatory disease activity and may be associated with delayed disability progression in a subset of patients with SPMS, particularly in those with ongoing inflammatory activity (143, 144) (143, 144). However, it is important to note that using NAT to treat RRMS increases the risk of developing progressive multifocal leukoencephalopathy. Treatment duration and JC virus antibody seropositivity can be used to stratify risk in patients receiving NAT, enabling more personalized discussions about the benefits and potential risks of treatment (145).

#### 3.1.3 GA

Glatiramer acetate (GA), a synthetic analog of myelin basic protein, was approved as an RRMS treatment in 1996. GA, which is a polypeptide mixture consisting of l-glutamic acid, l-lysine, l-alanine, and l-tyrosine, acts as an antigen associated with MS and can protect against myelin-responsive lymphocyte-induced neuroinflammation (117, 146–148). GA competitively binds to the major histocompatibility complex class II complex to block myelin antigen presentation to initial T cells. This GA binding also alters the differentiation of the initial T cells, causing preferential differentiation into Th2 cells, which arrive at the CNS. There, the Th2 cells secrete the anti-inflammatory cytokines IL-4, IL-5, IL-10, TGF-β, and IL-13, which have a protective effect against axonal injury and alleviate the pathologic process (**Figure 4**) (149, 150). In mechanistic studies, GA has been shown to promote the secretion of protective neurotrophic factors, such as insulin-like growth factor-1 (IGF-1) and brain-derived neurotrophic factor (BDNF). This effect has been well documented in EAE models as well as *in vitro* studies (151–153), in human studies, analyses of peripheral blood mononuclear cells from patients with MS have shown that GA treatment is associated with increased BDNF expression. This increase correlates with enhanced anti-inflammatory T-cell responses; however, a causal relationship between BDNF upregulation and clinical neurorepair outcomes remains to be further elucidated (154).

Studies indicate that GA treatment modulates both the number and functional state of dendritic cells and monocytes in patients with MS, promoting a more tolerogenic immune profile. Through alterations in antigen-presenting capacity and the cytokine milieu, these changes indirectly constrain pathogenic T-cell immune responses (155, 156). Concurrently, GA treatment has been associated with increased numbers of Tregs and enhanced suppressive function, contributing to the restoration of immune tolerance in MS (157, 158). Moreover, emerging evidence demonstrates that GA modulates B-cell antigen presentation, leading to reduced B-cell-mediated activation of CD4^+^ T cells and reshaping B-T cell interactions in MS (159).

GA delivery to EAE mice via nanocarrier liposomes, which have higher efficacy and longer duration of action and require less frequent administration, inhibited the inflammatory response and reduced areas of inflammation and demyelinating lesions in the CNS (160). Magnetic resonance imaging (MRI) examinations of 239 patients revealed that GA treatment caused a marked reduction in lesion count compared to an untreated control group, as well as a 33% decrease in relapse rates. GA administration also significantly reduced MRI-measured disease activity and burden (161). This therapy exhibits extremely mild side effects, indicating its potential to become a highly promising treatment option in the future (162). However, this drug exhibits significant interindividual variability in efficacy, with a substantial proportion of patients showing no or inadequate response to GA. Furthermore, there are currently no widely validated biomarkers (such as HLA typing, serum markers, or MRI features) capable of accurately predicting response prior to treatment.

#### 3.1.4 TER

**Figure 5:**
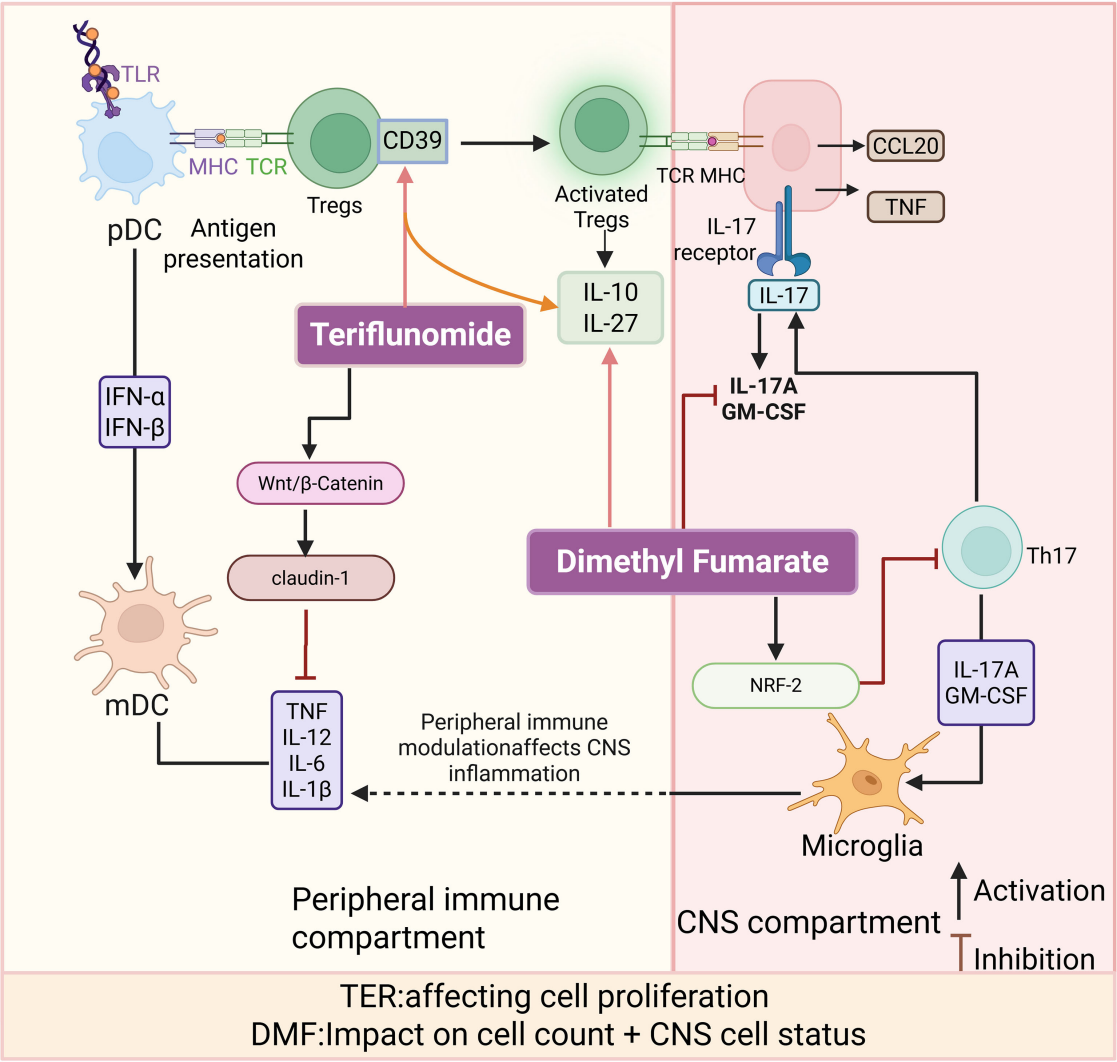
Pharmacological treatments targeting T-cell immunomodulation--DMF, TER. TER primarily acts in the peripheral immune compartment by limiting lymphocyte proliferation, enhancing regulatory T cells (Tregs) function, and modulating antigen-presenting cell activity, including plasmacytoid dendritic cells (pDCs) and myeloid dendritic cells (mDCs). TER-associated effects shown here include increased CD39 expression on Tregs, altered inflammatory cytokine production, and putative blood-brain barrier-related signaling involving Wnt/β-catenin and claudin-1. DMF reshapes peripheral immune responses while also exerting CNS-accessible effects through activation of nuclear factor erythroid 2-related factor 2 (NRF2), leading to reduced T helper 17 (Th17)-associated cytokines, including interleukin-17A (IL-17A) and granulocyte-macrophage colony-stimulating factor (GM-CSF), together with enhanced interleukin-10 (IL-10) and interleukin-27 (IL-27). Microglia are depicted within the CNS compartment as resident innate immune cells responsive to peripheral immune modulation. These mechanisms are associated with reduced relapse activity, lower MRI lesion burden, and delayed disability progression, with lymphopenia and infection risk as important safety considerations.

Teriflunomide (TER), a small molecule approved for the treatment of MS in 2012, inhibits dihydroorotate dehydrogenase (DHODH), a key enzyme in *de novo* pyrimidine synthesis. This pathway is required by CD4^+^ T cells and is expressed at high levels in proliferating T cells. The inhibition of DHODH expression inhibits CD4^+^ T cell proliferation, thereby limiting the involvement of T cells in neuroinflammatory processes (146, 163, 164). Reduced FOXP3^+^, a major regulator of regulatory Tregs, can be detected in MS patients (165). Tregs produce anti-inflammatory and tissue-repairing effects by inhibiting inflammatory factors, such as IL-6, IL-1β, IL-12, and TNF-α, which are activated and secreted by microglia and astrocytes, and by releasing anti-inflammatory factors, such as IL-10 (166–168). In human patients, TER administration promoted tolerogenic and suppressed pathogenic immune responses, as indicated by increased expression of the immunosuppressive marker CD39 on Tregs and decreased expression of the activation marker CXCR3 on CD4^+^ T helper cells (**Figure 5**) (169).

Beyond its well-established immunometabolic inhibitory effects, emerging evidence suggests that TER may exert additional regulatory actions by modulating BBB-associated signaling pathways. Specifically, experimental data indicate that TER can activate the Wnt/β-catenin signaling axis and is associated with upregulation of the tight junction protein claudin-1, a key structural component involved in maintaining BBB integrity and selective permeability (170). The Wnt/β-catenin pathway has been well documented to play critical roles in BBB development, repair, and regulation of tight junction protein expression (171, 172). However, it should be noted that the direct modulatory effects of TER on BBB integrity are currently supported primarily by mechanistic and correlative evidence, and their relative contribution to therapeutic efficacy in MS has not yet been clearly defined by prospective clinical studies. Beyond the well-established pathway-specific mechanisms, accumulating evidence indicates that TER treatment in MS is associated with coordinated immunophenotypic changes across both innate and adaptive immune compartments. Specifically, TER treatment has been linked to an increased frequency of CD39^+^ regulatory T cells, as well as a significant reduction in peripheral B-cell numbers in patients with MS (169, 173, 174). Collectively, these findings support the concept that TER exerts broader immunomodulatory effects that extend beyond its canonical antiproliferative activity.

After TER treatment, MS patients showed a significant reduction in MRI activity and a 55% reduction in newly enlarged T2 lesions. Similarly, TER treatment had a significant therapeutic effect on the combined index of MRI activity and clinical recurrence in 1,169 patients randomly assigned to a treatment group or control group. The annual relapse rate was lower in the treatment group than in the control group, and the treatment group showed a significant reduction in EDSS scores (175, 176). The most severe side effect following TER treatment is elevated serum aminotransferases, occurring in up to 50% of patients, which may subsequently lead to further liver damage. Other adverse reactions include hypertension, hair loss, diarrhea, and increased blood pressure (164, 177, 178).

#### 3.1.5 DMF

Dimethyl fumarate (DMF), approved by the FDA in 2013 for RRMS, exerts immunomodulatory effects primarily through activation of the nuclear factor erythroid 2-related factor 2(NRF2) antioxidant pathway in CNS cells (179–181). Treatment with DMF has been shown to suppress Th17 cell proliferation, inhibit the production of the IL-17A and GM-CSF, and enhance IL-10 secretion, particularly by attenuating IL-17 binding to astrocytes (182, 183). Sustained treatment further promotes regulatory T-cell activity and upregulates IL-27 expression, contributing to the attenuation of neuroinflammation in EAE models (**Figure 5**) (184). Human studies demonstrate reductions in effector memory and central memory T cells, as well as CD8^+^ T cells, accompanied by expansion of naïve T cells and downregulation of pro-inflammatory gene signatures (e.g., miR-155, HMOX1, OSGIN1) in monocytes (185–188).

Long-term follow-up over 13 years indicates sustained disease control, with a low annualized relapse rate (ARR ≈0.14), supporting its durable efficacy in RRMS (189, 190). The most common adverse events include flushing and gastrointestinal intolerance, while dose-dependent lymphopenia may occur and, if prolonged and severe, rarely predispose to progressive multifocal leukoencephalopathy (PML), necessitating regular monitoring of peripheral lymphocyte counts (191–193). Diroximel fumarate (DRF), a next-generation oral fumarate, demonstrates comparable efficacy with improved gastrointestinal tolerability (194, 195). Current understanding of DMF’s mechanism of action remains largely dependent on findings from *in vitro* cell culture studies. However, *in vitro* experimental systems inherently possess complexities: on one hand, DMF may undergo hydrolysis reactions during *in vitro* cultivation; on the other hand, methanol generated during or concurrent with the cultivation process may also produce independent biological effects within cells. Future clinical research is needed to conduct more in-depth investigations.

#### 3.1.6 S1P receptor modulators

Sphingosine 1-phosphate (S1P) receptor modulators are G-protein-coupled receptors distributed throughout the body (196). The U.S. FDA and the European Medicines Agency have approved four S1P receptor modulators-fingolimod, ozanimod, ponesimod, and siponimod-for the treatment of MS (146). S1P receptor modulators induce receptor internalization, ubiquitination, and proteasomal degradation, preventing lymphocytes from migrating toward inflammatory sites along S1P gradients. They also block lymphocyte efflux from lymph nodes by inhibiting transendothelial migration, ultimately reducing peripheral blood lymphocyte counts (197). However, the complete mechanism by which S1P receptor modulators exert their therapeutic effects remains unclear. The first-generation S1P receptor modulator fingolimod is a prodrug that requires phosphorylation to convert into its active form in order to gain affinity for the S1P receptor (198, 199). Second-generation S1P receptor modulators exhibit activity without requiring phosphorylation, enhancing their therapeutic potential for MS (200, 201). Compared to other DMTs, S1P receptor modulators may directly influence the CNS and cross the BBB, offering distinct advantages for MS treatment (202, 203). In the phase III trials, the annual relapse rate was significantly reduced in the fingolimod or siponimod treatment groups compared with placebo. Furthermore, the annual relapse rate was significantly reduced in the fingolimod, ozanimod, and ponesimod groups compared with the active control group (204–209). A study encompassing numerous clinical trials identified adverse reactions associated with S1P receptor modulators, including decreased lymphocyte counts, elevated liver aminotransferase levels, bradycardia and arrhythmias, macular edema, hypertension, recurrent herpes zoster, and convulsions (208).

#### 3.1.7 Alemtuzumab

Alemtuzumab is a humanized monoclonal antibody targeting the cell surface CD52 molecule, which is expressed on multiple cell populations including B and T cell and monocytes (210). It was approved in 2014 for the treatment of MS (211). Its unique mechanism lies in initiating an immune reconstitution process by depleting CD52-expressing lymphocytes (including T cells and B cells) from peripheral blood, thereby eliminating pathogenic T cell clones and restoring immune tolerance (210). CD52 is widely expressed on the surface of mature T cells. Upon drug binding, it can mediate T cell apoptosis through complement-dependent cytotoxicity (CDC) and antibody-dependent cellular cytotoxicity (ADCC) (212). Beyond this, alemtuzumab induces an asymmetric immune reconstitution process characterized by differential recovery kinetics among distinct immune cell subsets. Specifically, following alemtuzumab administration, B cells undergo rapid regeneration, whereas T cells-particularly memory CD4^+^ T cells-recover at a relatively slower pace. This disparity leads to long-term remodeling of regulatory and effector immune compartments. This phase of immune reconstitution is accompanied by a relative predominance of regulatory immune mechanisms, contributing to sustained disease control (213–215). However, imbalanced immune reconstitution, especially excessive B-cell regeneration, is considered closely associated with the development of secondary autoimmune complications.

Analysis of MS patients following alemtuzumab treatment revealed a marked increase in Tregs proportion, significantly suppressing the pathogenicity of Th1 and Th17 cells. Levels of IL-10, IL-27, and TGF-β showed sustained elevation, while IL-1β, IL-6, IL-17A, IL-12, and IFN-γ levels were suppressed (216, 217). The TOPAZ clinical trial and interim analysis of the TREAT-MS real-world study demonstrated that alemtuzumab maintained sustained efficacy in clinical and radiological outcomes among participants with highly active MS, with no new safety signals observed (218). It can be demonstrated that alemtuzumab possesses good safety and long-term efficacy. However, this therapy carries significant side effects, including a high risk of severe infections and autoimmune disorders, necessitating long-term monitoring of immune function following treatment (219–221).

#### 3.1.8 Cladribine

Cladribine is an adenosine analog prodrug that requires intracellular phosphorylation for activation (222, 223). It was approved in 2019 as an oral formulation for the treatment of MS. In quiescent cells, cladribine induces single-strand DNA breaks, leading to apoptosis; in proliferating cells, it induces cytotoxicity by disrupting DNA synthesis (224, 225). Cladribine preferentially targets lymphocytes, rapidly and persistently depleting both CD4^+^ and CD8^+^ cells, particularly preferentially eliminating self-reactive memory lymphocytes, while exerting a rapid but more transient effect on CD19^+^ B cells (226–228). Immunophenotyping studies demonstrate that cladribine significantly reduces Th1, Th17, and Tfh cells, with sustained suppression up to 24 months after treatment initiation, and shows a particularly pronounced effect on pathogenic IL-17, IFN-γ, GM-CSF effector populations associated with autoreactivity, accompanied by impaired T-B cell crosstalk (229–232).

Because cladribine is a small molecule capable of crossing the BBB and circulating in CSF, intermittent cladribine therapy can induce long-term remission in MS. This remission can persist without ongoing treatment. During treatment-free periods, the immune system regenerates and restores its ability to respond to infections, while MS disease activity does not resume (225, 233–236). Therefore, cladribine is considered an immune reconstitution therapy. Clinical studies indicate that cladribine can cross the BBB, achieving a concentration in CSF of approximately 25% of plasma levels, and rapidly distributes to tissues following administration (237). In a randomized, double-blind, multicenter study involving thousands of highly active RRMS patients, treatment with cladribine tablets significantly reduced the annualized relapse rate and markedly decreased the number of MRI lesions (235). The most serious adverse events associated with cladribine therapy include bone marrow suppression and secondary malignancies, as well as lymphopenia, headache, nasopharyngitis, and upper respiratory tract infections (238, 239).

#### 3.1.9 Anti-CD20

CD20 is a four-transmembrane phosphoprotein expressed on pre-B and mature B lymphocytes but absent on hematopoietic stem cells and terminally differentiated plasma cells (240, 241). Monoclonal antibodies targeting CD20 selectively deplete circulating B cells while largely preserving long-lived plasma cells, thereby reshaping adaptive immune responses without complete abrogation of humoral immunity (242, 243). In MS, the clinical efficacy of anti-CD20 therapy extends beyond simple B-cell depletion and critically involves modulation of pathogenic T-cell responses.

By presenting myelin antigens via MHC class II and providing costimulatory signals through CD80/CD86, B cells sustain activation and expansion of autoreactive CD4^+^ T cells, particularly Th1 and Th17 subsets. Depletion of CD20^+^ B cells diminishes antigen presentation capacity and attenuates costimulatory signaling, thereby limiting encephalitogenic T-cell activation. In parallel, disruption of germinal center dynamics impairs Tfh-B cell interactions, reducing amplification of autoreactive immune responses and constraining epitope spreading (241, 244). Anti-CD20 therapy also reshapes the cytokine milieu that governs T-cell differentiation. B-cell-derived IL-6, GM-CSF, and TNF-α, are reduced following treatment, leading to decreased Th17 polarization and attenuation of inflammatory T-cell responses within the CNS (245, 246). Longitudinal immunological analyses demonstrate a temporal shift in which B-cell depletion is followed by secondary modulation of T-cell activation states, supporting the concept that T-cell attenuation occurs downstream of B-cell targeting (246). Importantly, accumulating evidence indicates that a subset of pro-inflammatory CD20-expressing T cells exists in patients with MS. Both CD4^+^ and CD8^+^ CD20^+^ T cells have been identified, with CD20^+^ CD8^+^ T cells particularly associated with disease activity. Anti-CD20 monoclonal antibodies directly deplete these CD20-expressing T-cell populations, suggesting that therapeutic effects are not exclusively indirect but also involve partial direct targeting of pathogenic T cells (247–249).

Currently approved anti-CD20 monoclonal antibodies for MS include ocrelizumab, ofatumumab, and ublituximab, with rituximab widely used off-label (243, 250). Although these agents differ in epitope recognition and Fc engineering, leading to variable reliance on ADCC or CDC, their shared therapeutic principle lies in profound depletion of CD20^+^B cells and consequent modulation of T-cell-mediated neuroinflammation (251). Large randomized clinical trials have consistently demonstrated significant reductions in annualized relapse rates, MRI lesion activity, and disability progression in patients receiving anti-CD20 therapy (252–254).

Collectively, anti-CD20 monoclonal antibodies exemplify an indirect-T-cell-targeted strategy in MS. By eliminating pathogenic B-cell subsets, disrupting B-T cell crosstalk, reducing pro-inflammatory cytokine signaling, and depleting CD20-expressing T cells, these therapies rebalance the adaptive immune network and promote sustained suppression of autoreactive T-cell activity.

### 3.2 Traditional herbal therapy

Recently, traditional herbal medicine has garnered increasing attention. Curcumin has been reported to attenuate disease severity in EAE by inhibiting CD4^+^ T cells proliferation and suppressing Th1/Th17-associated inflammatory pathways. In addition, a small randomized clinical study in patients with SPMS evaluated a curcumin formulation (BCM-95) as an adjunctive therapy and demonstrated good tolerability, with MRI findings suggesting a potential effect on inflammatory disease activity (255, 256). Similarly, resveratrol has been shown in EAE models to modulate T-cell-mediated immune responses through suppression of Th1/Th17-related inflammatory signaling. Consistent with these findings, recent clinical studies in patients with MS indicate that resveratrol supplementation is well tolerated and may contribute to modulation of inflammatory immune activity (257, 258). Epigallocatechin-3-gallate (EGCG) has also been reported to regulate T-cell-mediated immune responses in EAE by inhibiting Th1/Th17 differentiation and reducing inflammatory cytokine production. Complementary clinical studies in MS patients suggest that EGCG-based interventions are safe and may exert immunomodulatory effects on inflammatory disease activity (259, 260). Furthermore, boswellic acids derived from Boswellia serrata have been shown to modulate T-cell-mediated immune responses by suppressing Th1/Th17-associated transcription factors (T-bet and RORγt) and inflammatory signaling while promoting Treg/Th2-related transcription factors (FOXP3 and GATA3). A clinical study in patients with relapsing-remitting MS further demonstrated that treatment with a standardized frankincense extract reduced MRI inflammatory activity and was associated with immunomodulatory effects on T-cell subsets (261–263). In addition, other natural compounds such as baicalein and paeoniflorin have shown anti-inflammatory effects in EAE models by modulating T cells responses and attenuating neuroinflammation (264, 265).

### 3.3 Novel therapeutics

Beyond currently approved DMTs, a growing body of research is exploring next-generation and experimental strategies aimed at achieving more precise immune regulation and durable disease control in MS. These emerging approaches include cell-based immunotherapies, such as CAR-T cell engineering and mesenchymal stem cell transplantation, as well as molecular interventions targeting non-coding RNAs and intracellular signaling pathways, including Bruton’s tyrosine kinase inhibition. In parallel, immune reconstitution strategies such as autologous hematopoietic stem cell transplantation are being actively investigated for their potential to reset immune tolerance and achieve long-term remission.

CAR-T cell technology enables autologous T cells to express chimeric antigen receptors, thereby directing the patient’s own immune system to precisely identify and eliminate target cells bearing specific antigens (266, 267). Recent studies have demonstrated that by engineering MOG-CAR regulatory Tregs to migrate to sites of inflammation following injection, the levels of IFN-γ secreted by Th1 cells were significantly reduced compared to controls. This indicates successful protection of CNS function alongside reduced systemic immunosuppression. This strategy leverages the innate capacity of Tregs to return to inflammatory tissues, thereby preserving central nervous function while limiting the breadth of immunosuppression (268). Furthermore, researchers injected engineered X-C motif chemokine receptor 1 (XCR1)-CAR-T cells into EAE mice, observing marked suppression of pathogenic Th1 cells and a significant reduction in clinical scores (269). The treatment of two MS patients using CD19-targeted CAR-T inhibited inflammatory relapse while removing CNS-resident B cells. CAR-T treatment for MS has already entered the experimental stage, and it may be widely used in the future for the treatment of MS after long-term evaluation of its safety and therapeutic efficacy (270, 271).

The use of mesenchymal stem cells (MSCs) has also received attention in MS research. MSCs are a group of non-hematopoietic stromal cells with broad capacity for immunomodulation, neurogenesis, and remyelination (272, 273). Motor deficits in female EAE mice were alleviated by intraperitoneal injection of MSCs, and molecular analysis revealed significant reductions in the expression of Th1 and Th17 cytokines and transcription factors (IFN-γ, STAT4, T-bet, IL-17, STAT3, and ROR-γt) (274, 275). IL-37, secreted by MSCs, blocked the transcription of pro-inflammatory cytokines and chemokines (e.g., IL-17, IL-1α, IL-6, TNF, and CXCL2), thereby ultimately reducing the pro-inflammatory effects of Th17 cells and attenuating the severity of symptoms in EAE mice, while transgenic expression of IL-37 attenuated inflammation through IL-1-R5/IL-1-R8 action (276–278). MSCs treatment induced FOXP3 expression through the secretion of indoleamine 2, 3-dioxygenase (IDO), which increased the proportion of Tregs in the spleens of patients with EAE, while the coculture of T cells and MSCs significantly upregulated FOXP3 expression in Tregs and increased the proportion of Tregs (279, 280). Thus, the use of MSCs could treat MS-related clinical symptoms through their effects on Th17/Tregs homeostasis.

The use of non-coding RNAs (ncRNAs), which include miRNAs, long ncRNAs (lncRNAs), and circular RNAs (circRNAs), is another area of interest in MS research. The ncRNAs function as regulators of gene expression. Although they do not encode proteins, they play key roles in gene regulation and cellular processes involving RNA molecules (281, 282). Many miRNAs are widely expressed in the CNS and immune system and perhaps can serve as markers in MS clinical practice in the future (283). Reports indicate that miR-92a mediates susceptibility to EAE via an intrinsic T-cell mechanism, by limiting the capacity of Tregs while simultaneously supporting Th17 pathogenic responses through direct suppression of the transcription factor Foxo1. This suggests that inhibiting miR-92a expression may represent a potential therapeutic target for MS (59). A study on miR-485 showed that it had an inhibitory effect on Th17-mediated responses in MS, downregulated STAT3, inhibited the inflammatory effects of Th17 cells, and attenuated the effects of EAE in mice (284). Moreover, recent findings indicate that IFN-β therapy suppresses miR-21-mediated Foxo1 to limit Th17 expression in MS patients. Consequently, inhibiting miR-21 may hold therapeutic potential for IFN-β non-responsive patient cohorts (119).

Bruton’s tyrosine kinase (BTK) is a key regulator governing the early maturation of B cells into memory and plasma cells, as well as B cell responses to antigen-stimulated proliferation (285). BTK inhibitors are small molecules classified as non-covalent reversible or covalent irreversible based on their binding mode to BTK (286). In the peripheral immune compartment, BTK regulates B-cell receptor signaling, antigen presentation, and co-stimulatory molecule expression, thereby influencing pathogenic T-cell activation and Th1/Th17-associated inflammatory responses (287–289). In contrast, within the central nervous system, BTK is expressed in microglia and other myeloid lineage cells, where it participates in innate immune signaling pathways (290). Preclinical and early-phase studies suggest that BTK inhibitors may modulate microglial activation and potentially support myelin repair, although evidence for remyelination in clinical settings remains limited (291, 292). Recent clinical data indicate that the BTK inhibitor fenebrutinib demonstrates favorable tolerability and exerts an early, potent, and sustained effect in limiting new focal brain lesions in patients with MS (293).

Autologous hematopoietic stem cell transplantation (AHSCT) is a multi-step procedure that destroys the immune system and allows it to rebuild from hematopoietic stem cells (294). On the one hand, AHSCT achieves long-term suppression of new focal inflammatory activity by first eliminating pathogenic T cells through immune reconstitution, followed by the restoration of immune tolerance via profound reconstruction of the immune system (295); on the other hand, AHSCT can suppress the inflammatory Th17 cell response, attenuate the expression of inflammatory cytokines, and further consolidate immune tolerance by promoting the expression of Tregs (296–299). Following AHSCT treatment, a broader range of new primitive T cells can be detected in the patient’s CSF or peripheral blood (300). A comparative clinical trial found that AHSCT was more effective than alemtuzumab and OCR in preventing relapse in MS patients, and suppressed MRI activity more significantly than alemtuzumab (301).

**Figure 6:**
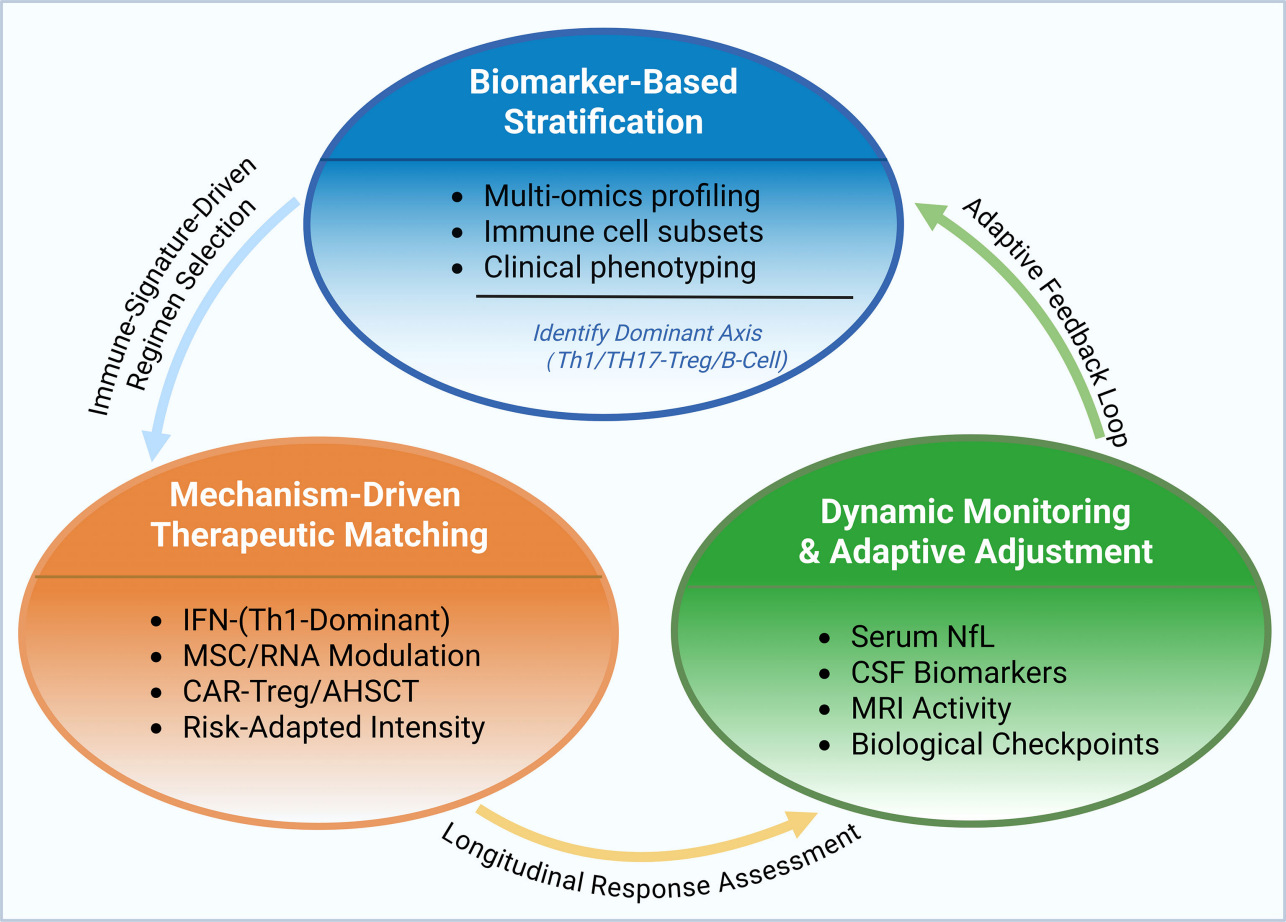
Conceptual framework-hypothesis-generating model Biomarker-based stratification (multi-omics profiling, immune cell subsets, and clinical phenotyping) identifies the dominant immune axis (T helper 1 [Th1], T helper 17 [Th17], regulatory T cell [Treg], B cell). This informs mechanism-driven therapeutic matching, including interferon-β (IFN-β; Th1-dominant), mesenchymal stem cell (MSC)/ribonucleic acid (RNA) modulation, chimeric antigen receptor Treg (CAR-Treg)/autologous hematopoietic stem cell transplantation (AHSCT), and risk-adapted intensity. Dynamic monitoring and adaptive adjustment are guided by neurofilament light chain (NfL), cerebrospinal fluid (CSF) biomarkers, magnetic resonance imaging (MRI) activity, and biological checkpoints, forming an adaptive feedback loop.

For precision treatment of MS, we propose a conceptual three-dimensional clinical framework consisting of biomarker-based stratification, mechanism-driven regimen matching, and dynamic monitoring with adaptive adjustment (**Figure 6**). This framework is intended to integrate current mechanistic knowledge and emerging therapeutic modalities into a coherent precision-medicine model, rather than to represent an already validated clinical algorithm. The stratification stage would incorporate multidimensional immune profiling, including genomic, transcriptomic, proteomic, and metabolomic signatures, together with immune cell subset analysis and clinical phenotyping (e.g., disease course, sex, age), to identify dominant immunopathological axes such as Th1-predominant, Th17/Tregs imbalance, or B-cell-driven inflammation (21). Therapeutic matching would then align intervention strategies with the predominant immune signature. For instance, IFN-β may be prioritized in Th1-dominant patients (302), while individuals with Th17/Tregs disequilibrium might benefit from MSCs-based approaches or RNA-targeted modulation (303). CAR-Tregs strategies or immune reconstitution therapies such as AHSCT could be considered for highly aggressive or progressive disease (270, 304). Importantly, treatment intensity and delivery methods may be individualized according to tolerability and risk profiles. Dynamic monitoring would rely on longitudinal integration of minimally invasive biomarkers (e.g., neurofilament light chain, NfL), CSF parameters, and MRI activity to construct individualized immune-response trajectories (21, 305–307). Predefined biological response checkpoints could guide escalation, de-escalation, or therapeutic switching, thereby minimizing systemic immunosuppression while maintaining disease control. This proposed framework aims to transform MS treatment from empirically sequenced therapy toward mechanism-informed, adaptive management. Nevertheless, prospective validation studies are required to determine its clinical feasibility and predictive accuracy.

## 4 Discussion

The pivotal role of T cell subsets in the pathogenesis of MS is now well recognized and has led to therapeutic strategies targeting distinct T cell populations. Pro-inflammatory Th1 and Th17 cells are considered key drivers of neuroinflammation and demyelination (52, 62), while Th2 and regulatory Tregs are mediators of anti-inflammatory and protective effects (53, 79). This functional distinction is reflected in current therapies, as IFN-β is invoked to suppress Th1 activity (118), GA to promote a protective Th2 shift (150), TER to restrict pathogenic CD4^+^ T cell proliferation while enhancing Tregs function (169), and DMF to trigger direct inhibition of pro-inflammatory Th17 pathways (186). Other therapies regulate the spatial distribution or availability of T cells, including NAT, which blocks VLA-4-mediated lymphocyte migration across the BBB, and S1P receptor modulators, which limit lymphocyte egress from lymphoid tissues (130, 197). In addition, immune-reconstitution therapies such as alemtuzumab and cladribine reshape T-cell populations through selective depletion and subsequent repopulation dynamics, while anti-CD20 therapies indirectly modulate autoreactive T-cell responses by disrupting B-T cell interactions (213, 229, 244). However, the interactions among T cell subsets within the complex immune microenvironment remain elusive. MS research often focuses on specific T cell populations, with the potential for the failure of therapeutic strategies targeting single cytokines. Furthermore, our current understanding of T cells remains largely confined to those associated with the peripheral immune system or cerebrospinal fluid, with no clear picture of their interactions with resident glial cells within the CNS. Unfortunately, the existing T-cell-targeted therapies typically affect the function of the entire immune system and fail to overcome issues of individual variability.

Currently, DMTs are regarded as the primary approach to addressing the unmet needs in MS management. However, a critical constraint persists: few DMTs can effectively cross the BBB without first targeting inflammatory lesions within the CNS, highlighting an urgent demand for novel therapeutics with inherent CNS penetration capabilities. Additionally, the high heterogeneity of MS symptoms and treatment-related adverse reactions associated with many DMTs continue to compromise patients’ quality of life, necessitating clinicians to individualize DMT selection based on disease phenotype, clinical presentation, and comorbidities. These limitations-insufficient CNS access, nonspecific immune modulation, and suboptimal tolerability-underscore the need to move beyond traditional DMT frameworks toward more precise and mechanistically targeted interventions.

Although these emerging therapeutic strategies show promise, they are also associated with significant limitations. CAR-T/CAR-Tregs therapies may lead to off-target effects, cytokine release syndrome, and neurotoxicity (308). MSCs carry risks of adverse immune reactions or tumorigenicity (309). BTK inhibitors are linked to infections, bleeding, and potential neurological toxicities (286), while AHSCT entails risks of transplant-related mortality, infections, and organ injury (310). From a manufacturing perspective, CAR-T/CAR-Tregs approaches require personalized cell processing, which is costly and prone to batch-to-batch variability. MSCs face challenges in source standardization and dose consistency, whereas AHSCT involves complex cell collection and intensive post-transplant care. Regulatorily, CAR-T, MSCs, ncRNA-based therapies, and BTK inhibitors remain at various stages of clinical investigation, with most not yet approved for routine use; AHSCT is currently restricted to highly selected, refractory cases. In terms of patient selection, CAR-T/CAR-Tregs therapies and AHSCT are generally reserved for severe disease forms, MSCs efficacy may be limited in advanced stages, and BTK inhibitors are contraindicated in patients with bleeding tendencies or active serious infections. Additionally, with most studies being small-scale and featuring short follow-up periods, there remains a lack of sufficient clinical evidence. Larger randomized controlled trials and long-term data are still needed to comprehensively evaluate the safety and efficacy profiles of these interventions.

Surprisingly, alongside the exploration of DMTs and novel precision therapies, certain traditional herbal medicines have demonstrated potential applications in managing MS. Preliminary research indicates that specific herbal formulations may play a role in alleviating motor dysfunction, sensory abnormalities, and emotional issues, as well as reducing relapse frequency (311). These formulations typically exhibit relatively low side effects and generally considered relatively affordable, rendering them attractive candidates for long-term disease management (312). However, akin to novel therapies requiring further clinical validation, research and application of traditional herbal medicines face significant bottlenecks. At the mechanistic level, the active constituents of the vast majority of herbs and their precise mode of action against T-cell-mediated neuroinflammatory cascades remain unclear (313). Existing evidence predominantly stems from small-scale studies, animal experiments, or retrospective case analyses, lacking validation through large-scale randomized controlled clinical trials. In practical application, herbal formulations suffer from inadequate standardization, challenging quality control, and inconsistent preparation processes. This undermines the stability of their components, the reproducibility of their efficacy, and their safety assurance (314). Consequently, for traditional herbal medicines to establish their place within the modern therapeutic framework for MS, they must undergo rigorous, large-scale studies to validate their efficacy, safety, and patient tolerability, much like novel therapies. Through continuous patient monitoring to assess their impact on neuroinflammation and myelin regeneration, and by innovatively integrating Eastern and Western diagnostic and therapeutic approaches, traditional herbal medicines may potentially carve out a more distinctive and accessible adjunctive treatment pathway for MS in the future.

## 5 Conclusion

This review summarizes the current knowledge regarding the immunological mechanisms of MS and highlights T cell-mediated dysregulation as being central to MS pathogenesis. Adaptive immunity in MS is collectively disrupted by genetic predisposition, environmental triggers, and gut microbiota imbalance, materializing largely as aberrant T cell polarization (Th1/Th17 hyperactivity and Tregs dysfunction). Pro-inflammatory cytokines (IFN-γ and IL-17) and impaired anti-inflammatory signaling (IL-10/TGF-β) drive chronic neuroinflammation. Emerging evidence implicates specialized cell subsets, as Th1-like cells sustain CNS inflammation, Tfh cells promote pathogenic B-cell responses, Tfr deficiency impairs regulation, and Th9/Th22 cells exhibit context-dependent effects. Current therapies that target T cells (e.g., IFN-β, GA) show limited CNS penetrance, underscoring the need for refined approaches, such as stem cell therapy or CAR-T. Future research should focus on neuroprotective strategies and multidisciplinary collaborations to address progressive neurodegeneration-the core challenge in MS treatment.
